# Differences in author ranking: Development of SIGAPS scoring system for Japanese version

**DOI:** 10.1002/jgf2.359

**Published:** 2020-07-09

**Authors:** Yuji Nishizaki, Yasuhiro Homma, Rieko Ueda, Patrick Devos, Shoji Sanada

**Affiliations:** ^1^ Medical Technology Innovation Center Juntendo University Tokyo Japan; ^2^ Department of Orthopedic Surgery Juntendo University School of Medicine Tokyo Japan; ^3^ Department of Cardiovascular Medicine Juntendo University Graduate School of Medicine Tokyo Japan; ^4^ Univ. Lille, CHU Lille, ULR 2694 ‐ METRICS: Évaluation des technologies de santé et des pratiques médicales Lille France; ^5^ Department of Medical Innovation Osaka University Hospital Suita Japan

## Abstract

Although the SIGAPS scoring system is extremely beneficial in evaluating the quality of academic papers and individual research achievement, values for author ranking may vary by country. If a SIGAPS scoring system outside of France is considered, the weighting by author ranking should also be reconsidered.

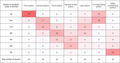

To the Editor,

The bibliometric software tool developed in France, namely Système d'Interrogation, de Gestion et d'Analyse des Publications Scientifiques (SIGAPS; “software to identify, manage, and analyze scientific publications”), can be used to calculate a new scoring system and evaluate the quality of academic papers,^1^ thus allowing assessment independent of research field. The SIGAPS is a superior scoring system that compensates for the shortcoming of Journal Impact Factor^®^ (IF) (Clarivate), which cannot be compared across research fields,^2^, ^3^ and is termed “relative Journal IF.” The SIGAPS is calculated by ranking Journal IFs from various research fields from high to low. Points are then assigned based on Journal IF percentiles.^4^

The SIGAPS components include the ranks of the journal and the author. The latter includes first or last author (4 points), second or second‐to‐last author (3 points), third author (2 points), or any other contributing authors (1 point) with a weighting factor.^4^ Although the SIGAPS scoring system is extremely beneficial in evaluating the quality of academic papers and individual research achievement, values for author ranking may vary by country. If a SIGAPS scoring system outside of France is considered, the weighting by author ranking should also be reconsidered. Therefore, we are trying to develop the scoring system from the Japanese perspective.

A questionnaire survey was conducted for principal investigators with registered clinical trials in the Japan Registry of Clinical Trials (jRCT). We sent a URL (Uniform Resource Locator) to access the questionnaire by e‐mail to 1567 researchers registered in jRCT as of June 1, 2019. This questionnaire was conducted using REDCap (Research Electronic Data Capture), and the researchers answered the questionnaire anonymously. The survey period ran from November 11 to 27, 2019. We asked the researchers about the levels of each author's contribution including first author, second author, third author, second‐to‐last author, last author, corresponding author, and other contributing authors.

A total of 39 researchers responded to the survey. Figure 1 shows the values of author ranking from high to low: “first author” > “corresponding author” > “second author” > “last author” > “third author” > “second‐to‐last author” > “other contributing authors.”

**Figure 1:**
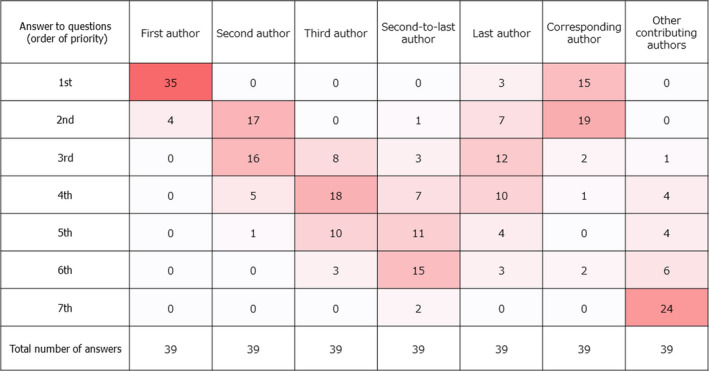
Results of questionnaire survey on author ranking. Numbers in the cells indicate number of responses, and the color depth of the cells is proportional to the number of answers

The values of the first and last authors are equally the highest in France,^1^, ^4^ whereas the corresponding and second authors are more valuable than the last author in Japan. Moreover, calculating the SIGAPS score overlooks the corresponding author in the French version, whereas the opposite is true for the Japanese version. The reason the value of the last author was not the highest in Japan could be as follows: The chairman or the professor tends to be the last author, regardless of their contribution to the research. In Japan, the funding issue is very critical, so the chief professor, who is primarily responsible for raising funds, occupies the last position instead of the primary conductors who could possibly be the next‐generation researchers.

Author ranking in the Japanese version largely differed from that of the French perspective. Point allocation for authors in the SIGAPS scoring system should be based on discussions with relevant stakeholders and according to the mindset of a country.

Finally, we were unable to obtain a sufficient number of responses because of the short survey period. The results of this study are preliminary, and it will be necessary to verify the results of this study by a large‐scale study.

## CONFLICT OF INTEREST

None.
